# Experiments on Temperature Changes of Microbolometer under Blackbody Radiation and Predictions Using Thermal Modeling by COMSOL Multiphysics Simulator

**DOI:** 10.3390/s18082593

**Published:** 2018-08-08

**Authors:** Yu-Zhen Deng, Shiang-Feng Tang, Hong-Yuan Zeng, Zheng-Yuan Wu, Der-Kuo Tung

**Affiliations:** 1 Department of Electrical and Electronic Engineering, Chung Cheng Institute of Technology, National Defense University, Taoyuan 33000, Taiwan; jcoh185@gmail.com (Y.-Z.D.); gto1244@gmail.com (H.-Y.Z.); shelter19811110@gmail.com (Z.-Y.W.); dktung@ndu.edu.tw (D.-K.T.); 2 Materials & Electro-Optics Research Division, National Chung Shan Institute of Science and Technology, Taoyuan 32599, Taiwan

**Keywords:** COMSOL Multiphysics, heat transfer model, temperature coefficient of resistance (TCR), microbolometer, blackbody, thermal conductance, thermal equivalent resistance differences (TERD)

## Abstract

In this study, we study a heat transfer model, with the surface of the microbolometer device receiving radiation from blackbody constructed using a COMSOL Multiphysics simulator. We have proposed three kinds of L-type 2-leg and 4-leg with the pixel pitch of 35 μm based on vanadium oxide absorbent membrane sandwiched with top passivated and bottom Si_3_N_4_ supporting films, respectively. Under the blackbody radiation, the surface temperature changes and distributions of these samples are simulated and analyzed in detail. The trend of change of the temperature dependent resistance of the four kinds of bolometer devices using the proposed heat transfer model is consistent with the actual results of the change of resistance of 4 samples irradiated with 325 K blackbody located in the front distance of 5 cm. In this paper, ΔT indicates the averaged differences of the top temperature on the suspended membrane and the lowest temperature on the post of legs of the microbolometers. It is shown that ΔT ≈ 17 mK is larger in nominal 2-leg microbolometer device than that of 4-leg one and of 2-leg with 2 μm × 2 μm central square hole and two 7.5 μm × 2 μm slits in suspended films. Additionally, only ΔT ≈ 5 mK with 4-leg microbolometer device under the same radiated energy of 325 K blackbody results from the larger total thermal conductance.

## 1. Introduction

The microbolometer is widely used in scientific field, defense and industries because of its high responsivity over a wide-bandwidth of electromagnetic spectra. A microbolometer senses the electromagnetic wavelengths on the basis of an electron-phonon interaction of the lattice upon electromagnetic irradiation, rather than electronic transition. Associated absorbed materials having larger temperature coefficient to resistance (TCR) defined as the relative resistance derivative 1/R dR/dT are preferred for bolometric application, i.e., for better responsivity it is desirable to have higher resistance change with small change in temperature [1,2]. In the last 20 years, the thermal imager based on uncooled microbolometer detectors has had much attention due to its light weight, low power consumption in ease of civilian and military applications [1,2,3]. High thermal isolation for the uncooled microbolometer detectors must be maximized to increase the sensitivity of the thermal detector. Besides good thermal isolation, a high TCR absorbed material such as VO_x_ or a-Si: H should be selected and good compatibility with standard Silicon-based semiconductor processes for high performance and a cost-efficient demand.

In this paper, the assessment of the significant performance for thermal sensitivity on VO_x_-based microbolometer detectors including four kinds of 35 μm-pitch device structure are proposed using the COMSOL Multiphysics Simulator incorporated with experimental results. The device operating principle is likely that the temperature-negative thermistor generated formed with the sandwiched membrane-like suspended film, which results in the temperature changes under thermal radiation to generate an electrical signal. Most of the design criteria of physical structures are having the slender double and four L-shaped legs for the suspension of thermal absorbent film and as much as possibly reduce thermal conductance to enhance their sensitivity. Undoubtedly, the devices must be placed in a vacuum-package environment to minimize the impact of ambient heat convection. For the confirmation of critical parameter optimization on the temperature sensitivity, responsivity, noise equivalent temperature difference (NETD) of VO_x_ microbolometer, it would be at no expense to the structure robustness and process complexity.

## 2. Physical Structure and Optoelectronic Parameters of Microbolometer

### 2.1. Schematics of Microbolometer Structure

**Figure 1:**
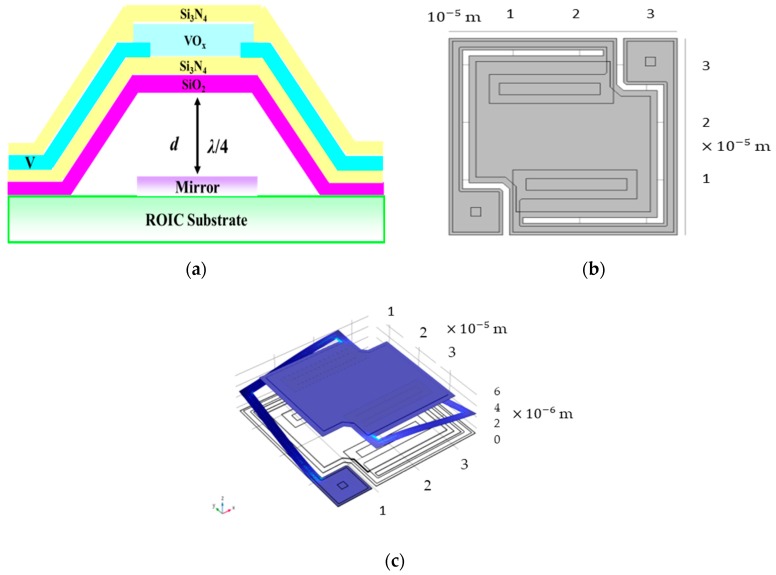
(**a**) Schematic of cross-sectional single-element microbolometer detector; (**b**) Top-view single-element microbolometer detector; (**c**) 3D single-element microbolometer detector.

The standard microbolometer structure consists of the suspended structure [4] i.e., sustained 200 nm-thick Si_3_N_4_/SiO_2_, 130 nm-thick absorbent VO_x_ and 100 nm-thick passivated Si_3_N_4_ sandwiched-like films, L-like legs for supporting the sandwiched-like films, where they are embedded with slender 12 nm-thick vanadium for electrical signal output, Al-mirror film beneath the suspended structure easily for the formation of Fabry-Pérot quarter-wave cavity resonator to enhance the absorbent thermo-efficiency shown in Figure 1a–c shows the 2D top-view and 3D schematics of standard 35 μm microbolometer structure, respectively. By using Equation (1), for normally zero order, we set *n* = 0, d can be simplified to λ/4*n*_r_, where *λ* is the peak-wavelength of absorbed infrared, and $n_{r}$ is refractive index of media in resonant cavity. Therefore, we have proposed the suspension plate supported by two or four L-shaped legs and the gap between bottom-sided suspended layer of SiO_2_ and the readout integrated circuit (ROIC) substrate is almost 2.5 μm to absorb peak wavelength of 10 μm. By the way, the thickness of SiO_2_ (130 nm) and Si_3_N_4_ (70 nm) are very thin with compared to the gap (~2.5 μm) in the resonator structure shown in Figure 1a. By using the mechanism of constructive interference on the bottom mirror and the top SiO_2_ of the microbolometer device, almost incident signals are fed back to the VO_x_ layer, thereby enhancing the performance of absorption. Thus, the gap between the suspended membrane and the metal mirror is designed to be ~2.5 μm.

(1) $$\;d=\frac{\lambda }{4n_{r}}+n\frac{\lambda }{2n_{r}}\;$$

where *n* = 0, 1, 2, …

Based on the material properties from Table 1, the electrical and optoelectronic characteristics are estimated analytically.

### 2.2. Thermal Characteristics and Optoelectronic Parameters of Microbolometer

In terms of heat-transfer in thermodynamics, the total thermal conductance ($G_{\mathrm{tot}}$) of microbolometer is written as:

(2) $$\;G_{\mathrm{tot}}=G_{\mathrm{leg}-\mathrm{tot}}+G_{\mathrm{gas}}+G_{\mathrm{radiation}}\;$$

where $G_{\mathrm{leg}-\mathrm{tot}}$, $G_{\mathrm{gas}}$ and $G_{\mathrm{radiation}}$ are thermal conductances of total legs, ambient environment and background radiation, respectively.

(3) $$\;G_{\mathrm{leg}-\mathrm{tot}}=2\times \left(\left(\lambda_{{\mathrm{Si}}_{3}N_{4}}\times \left(\frac{A_{\mathrm{leg}-{\mathrm{Si}}_{3}N_{4}}}{L_{\mathrm{leg}}}\right)+\left(\lambda_{{\mathrm{SiO}}_{2}}\times \frac{A_{\mathrm{leg}-{\mathrm{SiO}}_{2}}}{L_{\mathrm{leg}}}\right)+\left(\lambda_{v}\times \frac{A_{\mathrm{leg}-V}}{L_{\mathrm{legv}}}\right)\right)\right)\;$$

where $\lambda_{{\mathrm{Si}}_{3}N_{4}}$ and $\lambda_{{\mathrm{SiO}}_{2}}$ are the thermal conductivity of Si_3_N_4_ and SiO_2_; $A_{\mathrm{leg}-{\mathrm{Si}}_{3}N_{4}}$ and $A_{\mathrm{leg}-{\mathrm{SiO}}_{2}}$ are the cross-sectional area of Si_3_N_4_ and SiO_2_; $L_{\mathrm{leg}}$ is the total length of supporting legs; $G_{\mathrm{radiation}}$ is the thermal conductance generated from background radiation; σ is Boltzmann constant; ε is emissivity of radiation target; $A_{\beta }$ is absorbent surface area; T is temperature of radiation target.

(4) $$\;G_{\mathrm{radiation}}=8\times \sigma \times \eta \times A_{\beta }\times T^{3}\;$$

Normally, for the consideration of boundary in heat-transfer model, and the gap between the suspended membrane and substrate is only ~2.5 μm, smaller than 1 mm, the thermal conduction is dominated. Because *G*_gas_ is the thermal conductance attributes to thermal convection and conductance under the ambient environment, the value might be ignored. Taking Equations (3) and (4) into Equation (2), then $G_{\mathrm{tot}}$ could be derived shown in Equation (5). The *C*_tot_ is defined as Equation (6).

(5) $$\;G_{\mathrm{tot}}=2\times \left(\left(\lambda_{{\mathrm{Si}}_{3}N_{4}}\times \left(\frac{A_{\mathrm{leg}-{\mathrm{Si}}_{3}N_{4}}}{L_{\mathrm{leg}}}\right)+\left(\lambda_{{\mathrm{SiO}}_{2}}\times \frac{A_{\mathrm{leg}-{\mathrm{SiO}}_{2}}}{L_{\mathrm{leg}}}\right)+\left(\lambda_{v}\times \frac{A_{\mathrm{leg}-V}}{L_{\mathrm{legv}}}\right)\right)\right)+8\times \sigma \times \eta \times A_{\beta }\times T^{3}\;$$

(6) $$\;C_{\mathrm{tot}}=V_{1}\times c_{1}\times \rho_{1}+V_{2}\times c_{2}\times \rho_{2}\;$$

where symbol *c*, *ρ*, *V* are specific heat, density and volume of interested materials, respectively. *V* = $A_{\beta }\times h$, i.e., material area × thickness (normally called as height).

From Equation (6), the $C_{\mathrm{tot}}$ could be expanded as Equation (7).

(7) $$\;C_{\mathrm{tot}}=V_{{\mathrm{Si}}_{3}N_{4}}\times c_{{\mathrm{Si}}_{3}N_{4}}\times \rho_{{\mathrm{Si}}_{3}N_{4}}+V_{{\mathrm{SiO}}_{2}}\times c_{{\mathrm{SiO}}_{2}}\times \rho_{{\mathrm{SiO}}_{2}}+V_{{\mathrm{VO}}_{x}}\times c_{{\mathrm{VO}}_{x}}\times \rho_{{\mathrm{VO}}_{x}}\;$$

It is total thermal capacitance defined intuitively as Equation (8).

(8) $$\;C_{\mathrm{tot}}=C_{\mathrm{th}-{\mathrm{Si}}_{3}N_{4}}+C_{\mathrm{th}-{\mathrm{SiO}}_{2}}+C_{\mathrm{th}-{\mathrm{VO}}_{x}}\;$$

The thermal response time-constant (τ) is termed as Equation (9).

(9) $$\;\tau =\frac{C}{G}\;$$

Finally, Equation (2) to Equation (8) are substituted into Equation (10), then responsivity of microbolometer structure could be calculated.

(10) $$\;R_{\mathrm{espon}}=\frac{\mathrm{TCR}\times \beta \times \epsilon_{8-14}\times R_{\mathrm{eff}}\times I_{\mathrm{bias}}}{G_{\mathrm{tot}}\times \sqrt{1+\left(\omega^{2}\times \tau^{2}\right)}}\;$$

where β, $\epsilon_{8-14}$, $R_{\mathrm{eff}}$, *I*_bias_, ω, and TCR (also written as ∝) are fill-factor of pixel, absorbent ratio upon the infrared wavelength range of 8–14 μm, thermally effective resistance for the microbolometer, bias current into the two-terminal metal contacts, the modulated frequency and temperature coefficient of resistance, respectively. Furthermore, the temperature-dependent resistance is expressed as Equation (11):

(11) $$\;R\left(T\right)=R\left(T_{0}\right)e^{-\propto \left(\frac{T_{0}}{T}\right)\left(T-T_{0}\right)}\;$$

where T_0_ and T are initial ambient and thermal activated temperatures, respectively. In addition, effective thermal resistance is written as Equation (12), whereas the thermal bias current is defined as Equation (13)

(12) $$\;R_{eff}=\frac{R_{bol}\times R_{ROIC}}{R_{bol}+R_{ROIC}}\;$$

(13) $$\;I_{\mathrm{bias}}=\frac{U_{\mathrm{bias}}}{R_{\mathrm{bol}}}\;$$

while the temperature changes of microbolometer under the blackbody radiation ΔT, which is calculated as Equation (14)

(14) $$\;\Delta T=\frac{R_{\mathrm{bol}}\times U_{\mathrm{bias}}{}^{2}}{{\left(R_{\mathrm{ROIC}}+R_{\mathrm{bol}}\right)}^{2}\times G}\left(1-e^{-\left(\left(\frac{tr\times G}{C}\right)\right)}\right)\;$$

where *R*_bol_, *R*_ROIC_, *U*_bias_ and *t_r_* are resistance of microbolometer and ROIC, bias voltage upon the device and integrated time for the microbolometer.

## 3. Microbolometer Modeling and Experimental Setup

The experimental design is divided into three parts including the establishment of physical 2D, 3D microbolometer structure and measuring setup, thermal modeling of microbolometer under standard blackbody radiation, the measurement of optoelectronic parameters for being suitable with the proposed model. In Section 3.1, the four kinds of the geometric structure and their features are proposed for the performance assessment; the device modeling and the simulating process are shown in Section 3.2; the experiment setup with using thermo radiated blackbody on the four kinds of microbolometer structure is illustrated in Section 3.3.

### 3.1. Device Structures with 4 Kinds of 35 μm-Pixel Pitch

For the purpose of the optimization of optoelectronic features, the four kinds of microbolometer structures are designed, shown in Table 2 with the different physical parameters and geometric sizes in detail, where Design 0 is the standard L-shaped dual-leg structure for reference with previous paper published [5,6]; Sequentially, Design 1 to 3 are modified types. The lengths of Si_3_N_4_/ V in each leg of Design 0, Design 1 and Design 3 are 66/60 μm, only 33/30 μm for each leg of 4 legs in Design 2. Design 1 with 2 μm × 2 μm central square hole and two 7.5 μm × 2 μm slits on the suspended area is designed for increasing the process speed of removal polyimide and having some helpful result for platform stress relief in order to decrease the risk in collapse of suspended platform; Design 2 with 4-leg structure used for strengthen the suspended supporting while the polyimide is removed; Design 3 is the structure with increasing the gap between two contacts, from 14.5 to 20.5 μm (i.e., increasing active response region) for increasing the response signal of device irradiated from the target, respectively. Apart from above modified physical structures, the issue on stress or deformation of suspended membranes and 2 and 4 supporting legs of Design 0 to Design 3 are even concerned of interest. However, for the distinguishing physical differences mentioned above and performance assessment attributable to the variables, the other structural parameters of the 4 kinds of microbolometer are designed as the same, such as pixel pitch, size of suspended platform, width of each leg, width of each electrode in the leg, width of each electrode on the platform, width of each VO_x_ contact, length of each VO_x_ contact, thickness of Si_3_N_4_, thickness of VO_x_ film, thickness of V film are 35 μm, 28 μm, 2 μm, 1 μm, 2 μm, 4 μm, 26 μm, 70 nm, 130 nm and 12 nm, respectively.

**Figure 2:**
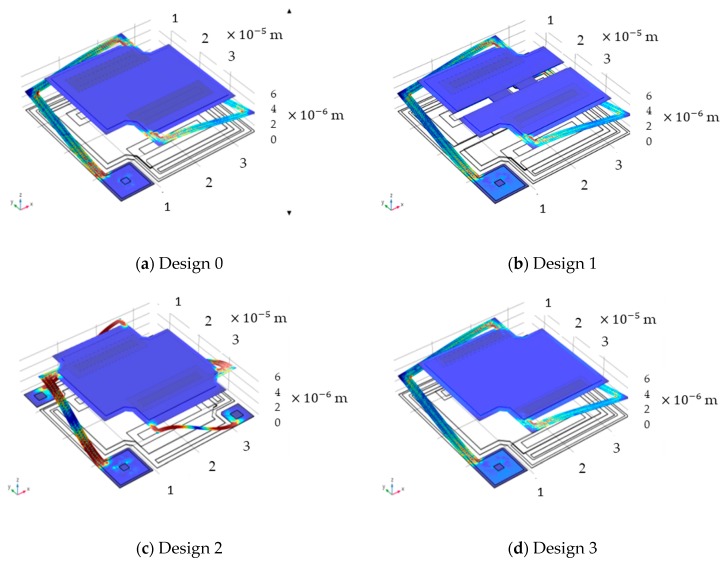
The 4 kinds of top-view 3D geometric 35 μm pixel pitch single-element microbolometer structures, where (**a**) Design 0 is the standard L-shaped dual-leg structure for reference; (**b**) Design 1 with 2 μm × 2 μm central square hole and two 7.5 μm × 2 μm slits on the suspended area; (**c**) Design 2 with 4-leg structure used for strengthen the suspended supporting and (**d**) Design 3 is the structure with increasing the gap between two contacts, from 14.5 to 20.5 μm.

Figure 2 shows the 4 kinds of 3D geometric single–element microbolometer structures based on Table 2 and using expanded from COMSOL 2D drawing toolbox. From the observation of Design 0, 1 and 3, there could be only 2 L shaped legs to support the suspended membrane except for Design 2 with four legs for the robust of suspended infrared films. The intrinsic stress tensors of each suspended film incorporated with supporting legs are considered referred to Table 1 to simulate the stress distribution of 3D physical structures by COMSOL 3D simulator tool. From Figure 2 to the conclusion, it is very clear to show that the great low deformation and high uniformity stress on the suspended membranes of four kinds of microbolometer structures. In particular, the higher stresses (colored in lighter blue and even yellow-red or white) happen in the interfaces between posts and lower parts of L-shaped legs as well as suspended membrane and topper parts of the legs. The fracture would be easily generated on the interface sections. Something twisting results in bending deformation on the middle region of the supporting legs in order to keep torque balance of overall unit-cell system.

Based on the device structures in the previous Figure 2 and Table 2, the optoelectronic parameters and thermal distribution on the surface of microbolometer are calculated and simulated under the same temperature—radiation condition.

### 3.2. Thermal Modeling Process on 4 Kinds-of the Microbolometer Structures

**Figure 3:**
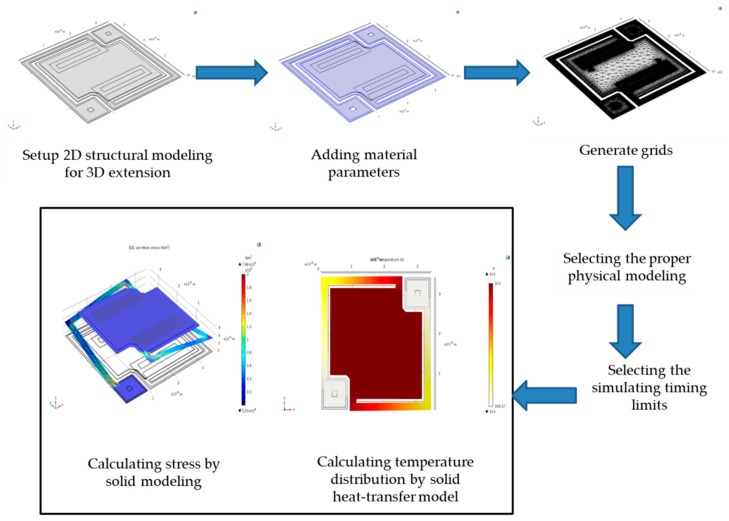
Setup processes of modeling of single-element suspended microbolometer.

Firstly, using the finite-element analytic method by COMSOL [7,8,9,10], the 3D physical microbolometer structure is modeled. The following steps include 2D top-view drawing of each physical layer built in microbolometer device, the set-up of dimensional size and thickness of each layer, inputting the material parameters of each layer deposited for microbolometer device, structuring proper meshed grids on single device element, and combining the heat-transfer physical equation modeling to solve the convergent results of thermal distribution on the suspended microbolometer device. For decreasing the calculated loading of central processing unit (CPU) and getting simulating times, structure stress distribution of each layer is excluded. The modeling processes are followed as step by step, shown in Figure 3:

1. 2D geometrical structure modeling for 3D extension with the suspended device structure.
2. Adding the materials of bolometer structure involved such as SiO_2_, Si_3_N_4_, vanadium oxide and vanadium metal.
3. Using finite element method to generate the grids for the solving the optimal 3D material stress distributions in the proposed bolometer based on suspended structure efficiently.
4. For simplifying the calculating complexity, the analytic modeling is introduced using geometric elastic stress under timing-imitated mode.
5. Sequentially calculating the temperature distribution on the surface of suspended structure by solid heat-transfer model under illuminated from flat-plate typed black-body radiation and vacuum condition.
6. Finally, the 3D stress and structure temperature distributions would be solved to be made for the performance optimization of single bolometer pixel independently.

**Figure 4:**
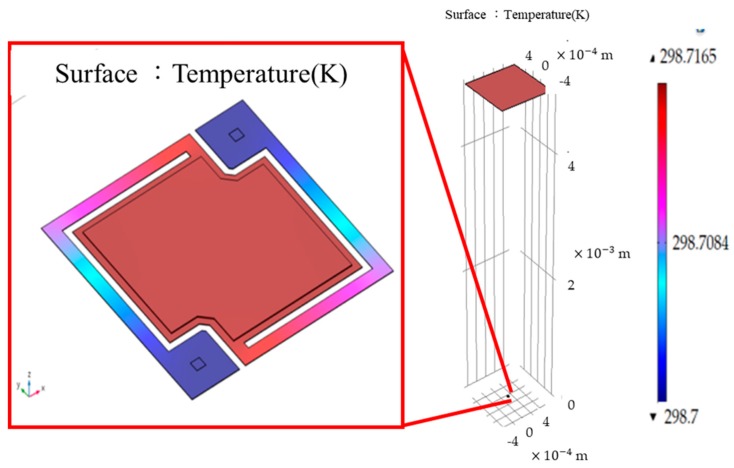
The simulated architecture for the illuminated microbolometer with pixel-pitch of 35 μm.

Moreover, the thermal distribution of the single-element microbolometer is simulated and characterized. The flat plated incident thermal source under the constant temperature illuminates upon the surface of single element suspended structure is shown in Figure 4. Due to considering the CPU efficiency and simulated time limitation, the two contacts of vanadium on VO_x_ and vanadium sandwiched in legs are ignored under calculating thermal distribution using 3D heat-transfer modeling. The other three different bolometer structures are illuminated using the same experimental design as Design 0 (i.e., referred structure) where the effect for thermal expansion on these devices is also neglected. The temperature of 323 K for the 5 cm × 5 cm flat-plate blackbody source is setup. The incident distance of blackbody to the surface of microbolometer is 5 cm. The same heat flux density incident uniformly to the surface of device with pixel-pitch of 35 μm under vacuum condition is assumed. The temperature of legs attached on substrate is 298 K under thermodynamic stead-state.

### 3.3. The Experimental Setup for Thermal Radiated Microbolometer Illuminated under Blackbody and Vacuum Condition

**Figure 5:**
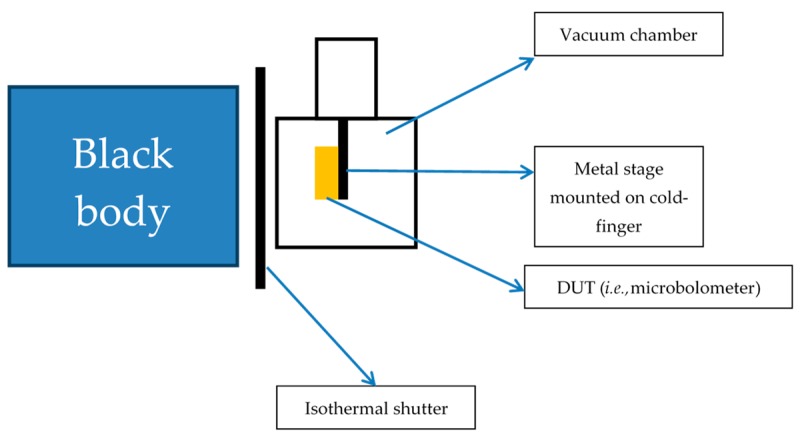
The experimental setup for the thermal radiation on the 4 different microbolometer structures.

In Figure 5 it is shown that the experimental setup is used for the deduction the proposed thermal modeling previously. The experimental steps are as follows:

- Put the device under test (DUT) on the stage mounted on cold-finger under vacuum chamber with pumping.
- Setup the blackbody temperature of 323 K illuminated or blocked with isothermal shutter.
- Using high precise semiconductor analyzer (HP 4156 B, Agilent Technologies, Englewood, CO, USA) with sampling mode to provide the constant current of 1 μA on the DUT, the voltage signals are measured. Because treated with small direct current (DC), the self-heating of DUT could be ignored.
- During the full measured period of 90 seconds the isothermal shutter in former 30 seconds is blocked in front of DUT, later opening the isothermal shutter and blackbody source illuminating into DUT for 30 seconds, finally blocking the radiated source of blackbody for 30 seconds.
- Based on above process, the measured steps are the same for the 4 proposed DUTs and the ΔR might be extracted under the temperature of 323 K from blackbody radiation.
- Finally, the 3D stress and structure temperature distributions would be solved to be made for the performance optimization of single bolometer pixel independently.

## 4. Results and Discussions

Based on the analytical equations in Section 2.2, the testing measurements for main functional parameters and published paper previously, the optoelectronic characteristics of 4 different types of microbolometer are achieved in Table 3. From above results, the different thermal responsivities (*R*_espon_), response times (τ), absorbed ratio (*η*) and NETD are obtained while changing the active absorbed size scale and device structures. It leads to the main reasons for the variety of microbolometer performance [8,9]. D2 has the largest thermal-conductance (1.57 × 10^−9^ W/T) because of the four supporting legs more than two legs of the other three kinds of microbolometers. D0 is standard structure with pixel-pitch of 35 μm. Based on Design 0, regarding Design 1 and Design 2, it decreases the process complexity and increases the device performance. Besides, adding the excess two legs of microbolometer, it can be stress-relieved easily for improving process yield. Nevertheless, for Design 2 under the limitation of same pixel-pitch size, having shortened the absorbed area results in lower responsivity and detectivity. By contrast in Design 0, Design 3 extending the gap between top vanadium contacts on VO_x_ leads to enlarging the absorbed area of incident thermal signal. Therefore, it increases by almost two times the responsivity compared with Design 0 and possesses the highest absorbed ratio (*η*) among the four proposed device structures. The noise equivalent power (NEP) of Design 3 is lower than that of Design 2 because of less leg numbers. Except high response time for Design 2 with four supporting legs, the critical parameters of the other three device structures are superior to that of Design 2. To conclude, extended the gap of top two vanadium contacts is the best design structure while it is only considered for optimizing optoelectronic performance and ignored for process effects and yield issues.

**Figure 6:**
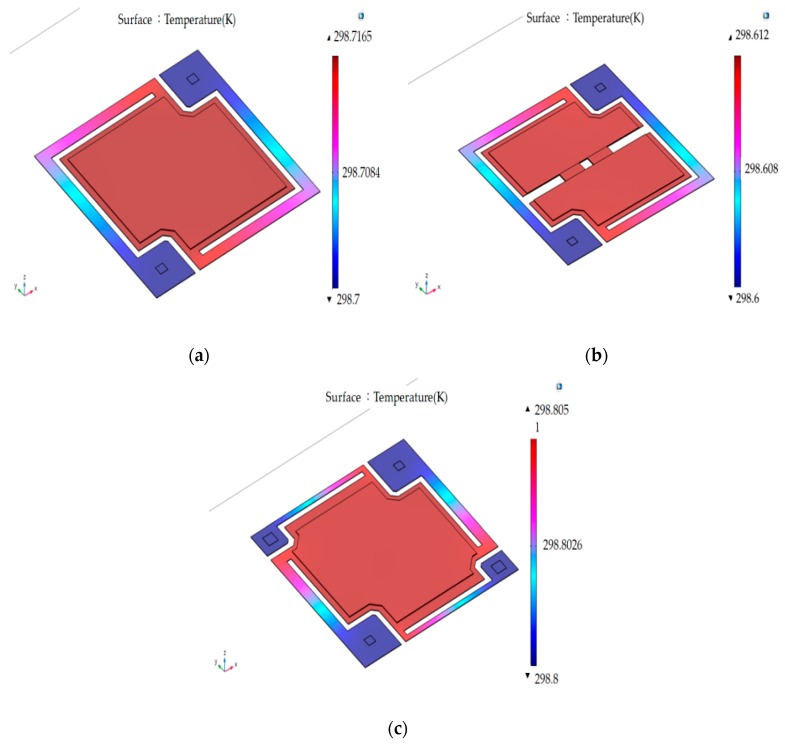
Thermal distribution of the surface of (**a**) Design 0 and Design 3; (**b**) Design 1 and (**c**) Design 2 under the same illuminated condition.

Based on the modeling processes and initial condition of experimental design in Section 3.2, the average temperatures of suspended film and the substrates of the microbolometer sunder the 3D thermal modeling are 298.717 (Design 0 and 3), 298.612 (Design 1) and 298.805 mK (Design 2). In Figure 6, the temperature differences are 16.5, 12 and 5 mK between the surface of the suspended microbolometers and the substrates, respectively. It is obvious that the above value of Design 2 is the smallest among the 4 microbolometer structures due to the largest thermal conductance shown in Table 3.

Under thermodynamic steady-state condition, the temperature differences (ΔT) between the suspended film and the substrates of the microbolometers are described. These are to indicate the averaged differences between the top temperature on the suspended membrane and the lowest temperature on the post of legs of the microbolometers and shown in Equation (15) [10], where W_i_ is the incident energy power. From the equation, ΔT is positively related to the absorbed area (VO_x_ thin film) and *W_i_*, and inversely proportional to the thermal conductance (G) of the microbolometer.

(15) $$\;\Delta T=\frac{A_{d}\times W_{i}}{G}\;$$

Using the material coefficient taken from Table 1, Table 2 and Table 3 and combining the incident energy power from the temperature of 323 K flat-plated blackbody, the analytic temperature difference ΔT could be obtained. Subsequently, substituting into Equation (10). The resistance differences (Δr) are also achieved from $R\left(T\right)-R\left(T_{0}\right)$ in Equation (11). The thermal equivalent resistance differences (TERD i.e., Δr) are calculated and shown in Table 4. The 3D modeling for thermal distribution [11] on the surface of microbolometer in Figure 6 and using analytic ΔT in Equation (15) have a similar trend. It means that they can be cross-validated each other.

For the propose of verification of thermal equivalent resistance differences based on Equation (15), the proposed experiment is shown in Figure 6 and described in Section 3.3 in detail. The ΔV and ΔR/ΔT are measured and calculated shown in Table 5. The electrical property of VO_x_ suspended film is similar to negative temperature-like resistance.

**Figure 7:**
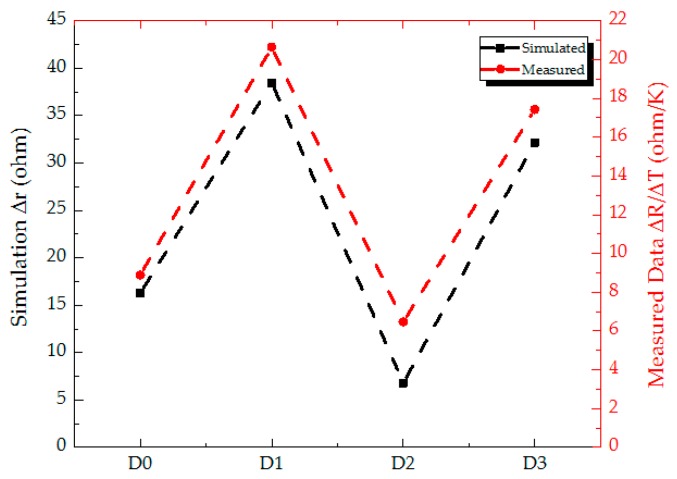
The ΔR/ΔT and Δr are measured and simulated, respectively.

ΔR/ΔT measured shown in Table 5 has high-order positive relation to Δr converted from ΔT taken with 3D thermal modeling, and its related plot is shown in Figure 7. In this figure, the black and red dashed lines indicate the Δr (simulated) and ΔR/ΔT (measured) extracted from Table 4 and Table 5, respectively. In all of 4 kinds of proposed microbolometer structures, Design 1 has the largest Δr and ΔR/ΔT. It can be explained the thermal capacitance of Design 1 is the smallest contrasted to the other structures and results in lower τ to increase the responsivity of the microbolometer structure referred to Equation (10). On the other side, Design 2 has the smallest Δr and ΔR/ΔT. It is due to the largest thermal conductance of Design 2 with four legs among four kinds of microbolometer structures, which lead to the lowest responsivity. With compared to Design 0 and Design 3, Design 3 increases the spacing from 14.5 (standard) to 20.5 μm between two vanadium contacts for the propose of increasing responsivity and detectivity. From Table 3, the values of responsivity and detectivity of Design 3 are calculated using the analytic equations in Section 2.2 incorporated with the physically geometry structures and the associated material coefficients to approach 1.25 × 10^5^ V/W and 1.65 × 10^9^ cm-Hz^1/2^/W, respectively. The above values of Design 3 have almost two times of Design 0 (standard). The trend is also deduced to cross-prove the credibility by the analytic equations and thermal distributed modeling. It is noted that the modeling proposed with COMSOL Multiphysics Simulator can predict the parametric performance of different kinds of microbolometer structures in advance. Furthermore, the evaluation of device structures associated with optoelectronic parameters is also a very significant for subsequent device processes.

## 5. Conclusions

At first, the output voltages of microbolometer devices are measured with the constant current of 1 µA while shutter is under shielding and opening, respectively. Secondly, the differences of output voltages are obtained. In the meantime, the differences of resistances are calculated with Ohmic law. Then the ratios of differences of the resistance and the temperature (ΔR/ΔT) are derived. For the evidence of credibility of the thermal distribution upon the four proposed microbolometer structures, COMSOL simulator is used with thermal modeling for the achievement of distribution of temperature on the top surface of microbolometer device, e.g., the simulated result of D0 in the one of four structures. The averaged ΔT are taken accompanied with known ∝ and R(T0) to be substituted. The resistance differences (Δr) are achieved from $R\left(T\right)-R\left(T_{0}\right)$, i.e., TERD. The experimental (ΔR/ΔT) and simulated (Δr) results are obtained and well cross-evidenced. The above data is illustrated in Section 4. The above curve trends from D0 to D3 are the similar and the proposed modeling is satisfied well while the simulated condition must be consistent with experimental setup (e.g., the values of ∝ and R(T0)) as far as possible.

It is obvious that the value of ΔT in four-leg structure is the smallest due to the largest thermal conductance, although it is expected highest yield under the process of polyimide removal among the four microbolometer structures. The microbolometer structures are in a vacuum environment, and the temperatures of their suspended layers and legs are setup in room temperature initially, and then illuminated by the blackbody with the temperature of 323 K under the steady-state thermodynamic condition. Accompanying with COMSOL simulator is used with thermal modeling for the achievement of distribution of temperature on the top surface of microbolometer device.

By using the COMSOL Multiphysics Simulator, we construct 3D thermal modeling, which can simulate the thermal distribution on the microbolometer device with pixel-pitch of 35 μm. Analytic heat-transfer equations and experimental measurement of devices illuminated from blackbody source under the thermo-dynamic steady-state condition can be evidence to its credibility as well. From the proposed construction of optoelectronic parameters, 3D thermal modeling and the analytic heat-transfer equations with pixel-pitch of 35 μm, the evaluation of microbolometer performance under the effect of ambient environment is implemented well in advance. With regard to designer for the development of novel microbolometer structure, the usage and pre-assessment with the physical parameters and 3D thermal modeling effectively reduce the cost of device process and experimental destructive risk. In the future, the 3D thermal modeling will be incorporated with stress modeling under the considering the structure resonance on the suspended plate of microbolometer device. The numerically and physically composite modeling would be achieved precisely to fit physical device according to the emulated consideration.

## Figures and Tables

**Table 1:** Material characteristic parameters of suspended and sandwiched structure for Microbolometer detector.

Material	Thermal Conductivity (W/cm·K)	Thermal Capacitance (J/kg·K)	Density (g/cm^3^)	Resistivity (Ω cm)	Young Modulus (GPa)	Poisson’s Ratio
SiO_2_	1.4	787	2.196	~10^23^	75	0.17
Si_3_N_4_	0.02	3.33	2.3	~10^15^	300	0.26
VO_X_	0.05	5.00	4.34	1	80	0.24
Vanadium	3.07	489	6	1.97 × 10^−^^7^	128	0.37

**Table 2:** The significant differences in specifications of design 0 to 3 device structure for microbolometers.

Parameter	Design 0 (D0)	Design1 (D1)	Design 2 (D2)	Design 3 (D3)
Number of legs	2		4	2
Si_3_N_4_/V in each leg length (μm)	66/60		33/30	66/60
Leg shape (μm)	L	L	I	L
Vacancy on platform	n/a	Center: 2 μm × 2 μm Slits: 2 μm × 7.5 μm	n/a	n/a
Spacing of electrodes on the platform (μm)	14.5			increase the space from 14.5 (standard) to 20.5

**Table 3:** Optoelectronic performance parameters of Design 0 to 3.

	Unit	Design 0 (D0)	Design 1 (D1)	Design 2 (D2)	Design 3 (D3)
Response time	(mS)	6.96	6.59	2.73	6.02
*G*	W/T	7.9827 × 10^−10^	7.72692 × 10^−10^	1.57 × 10^−^^9^	8.0516 × 10^−10^
Responsivity	V/W	6.899 × 10^4^	1.22614 × 10^5^	2.8306 × 10^4^	1.25148 × 10^5^
Detectivity	$\frac{{\mathrm{cmHz}}^{1/2}}{W}$	9.11595 × 10^8^	1.6092 × 10^9^	3.7369 × 10^8^	1.64653 × 10^9^
NEP	W	3.839 × 10^−12^	2.1749 × 10^−12^	9.366 × 10^−12^	2.1257 × 10^−12^
*η*%	-	6.615	9.474	5.338	11.347
NETD	mK	99.219	39.24	299.962	32.028

**Table 4:** Thermal equivalent resistance differences (TERD, i.e., Δr) of 4 proposed microbolometer structures.

	Design 0 (D0)	Design 1 (D1)	Design 2 (D2)	Design 3 (D3)
Thermal equivalent resistance differences Δr (Ω)	16.291	38.448	6.802	32.160

**Table 5:** ΔV and ΔR/ΔT of 4 proposed microbolometer structures are measured.

	Design 0 (D0)	Design 1 (D1)	Design 2 (D2)	Design 3 (D3)
ΔV (mV)	0.209	0.528	0.156	0.448
ΔR/ΔT (Ω/K)	8.886	20.64	6.475	17.43
